# The *Dictyostelium discoideum acaA* Gene Is Transcribed from Alternative Promoters during Aggregation and Multicellular Development

**DOI:** 10.1371/journal.pone.0013286

**Published:** 2010-10-11

**Authors:** Maria Galardi-Castilla, Ane Garciandía, Teresa Suarez, Leandro Sastre

**Affiliations:** 1 Instituto de Investigaciones Biomédicas, Consejo Superior de Investigaciones Cientificas/Universidad Autónoma de Madrid (CSIC/UAM), Madrid, Spain; 2 Centro de Investigaciones Biológicas, Consejo Superior de Investigaciones Cientificas (CSIC), Madrid, Spain; University of Birmingham, United Kingdom

## Abstract

**Background:**

Extracellular cAMP is a key extracellular signaling molecule that regulates aggregation, cell differentiation and morphogenesis during multi-cellular development of the social amoeba *Dictyostelium discoideum*. This molecule is produced by three different adenylyl cyclases, encoded by the genes *acaA*, *acrA* and *acgA*, expressed at different stages of development and in different structures.

**Methodology/Principal Findings:**

This article describes the characterization of the promoter region of the *acaA* gene, showing that it is transcribed from three different alternative promoters. The distal promoter, promoter 1, is active during the aggregation process while the more proximal promoters are active in tip-organiser and posterior regions of the structures. A DNA fragment containing the three promoters drove expression to these same regions and similar results were obtained by in situ hybridization. Analyses of mRNA expression by quantitative RT-PCR with specific primers for each of the three transcripts also demonstrated their different temporal patterns of expression.

**Conclusions/Significance:**

The existence of an aggregation-specific promoter can be associated with the use of cAMP as chemo-attractant molecule, which is specific for some *Dictyostelium* species. Expression at late developmental stages indicates that adenylyl cyclase A might play a more important role in post-aggregative development than previously considered.

## Introduction

Intercellular communication plays a central role in multi-cellular development, coordinating processes such as cell migration, proliferation and differentiation that are the basis for morphogenesis. One of the simplest organisms where these processes have been studied is the social amoeba *Dictyostelium discoideum* (for recent reviews, [1], [2]). These organisms live as individual amoebe in forest soils. Upon starvation, up to 100.000 cells aggregate together and form a fruiting body, composed of a basal disk, a stalk and, on top of it, a sorus.

*D. discoideum* fruiting body formation is one of the more primitive examples of multi-cellular development but, even so, is a complex process that is tightly regulated [3]. Cell aggregation is mediated by cAMP in *D. discoideum* [4]. Cells in the aggregation fields are able to move towards increasing cAMP concentrations and to secrete cAMP, so that the signal gets amplified [5]. Upon aggregation, mounds are formed where cells differentiate in two main alternative pathways: prestalk or prespore cells. Prestalk cells associate together and move to the upper part of the mound, where they emerge as a tip [6], [7], while prespore cells remain in the lower part of the mound [8]. The tip region acts as an organizing center during later development [9], [10]. Culmination takes place when prestalk cells, located in the tip, migrate towards the substrate through the mass of prespore cells, elongate and synthesize a cellulose outer layer. As a consequence, prespore cells are raised from the substrate to form a sorus, and complete the differentiation process.

Several signaling molecules coordinate *D. discoideum* development but, among them, cAMP plays a central role (reviewed in [1], [11]). As mentioned above, extracellular cAMP first mediates aggregation [4]. Later on, cAMP secreted from the tip is required for prestalk and prespore cells sorting in the mound [12]. Extracellular cAMP at a high, constant level is required for in vitro prespore and prestalk differentiation [13], [14], inducing or repressing the expression of cell-type specific genes [15]. The decision of initiating culmination is also dependent on extracellular cAMP, that activates the STATa transcription factor at the tip organiser region, in the anterior of the slug, initiating a regulatory cascade that proceeds through activation of the CudA transcription factor [16], [17] and the tip-organiser-specific expression of their targets genes, such as *expl7* [18]. Finally, high extracellular cAMP levels are required for spore differentiation [19], for GSK-3 mediated inhibition of stalk cell formation [20], [21] and for regulation of spore germination [22]. Additional signaling molecules contribute to coordinate some of these processes. For example, stalk cell differentiation is induced by the chlorinated hexaphenone DIF-1, secreted by prespore cells, in addition to cAMP, as shown in in vitro experiments [23]. Similarly, spore differentiation is quickly induced by SDF1 and SDF2, produced by prestalk cells, in the presence of high levels of extracellular cAMP [24]. cAMP also plays an important role as an intracellular signaling molecule. For example, cAMP-dependent activation of protein kinase A is required for cAMP signaling at initiation of development [25] and spore differentiation [26], [27].

The extensive use of cAMP as signaling molecule requires a tight control of its synthesis and degradation (reviewed by [11], [28]). Synthesis is catalyzed by three different adenylyl cyclases, encoded by the genes *acaA*, *acrA* and *acgA*. Degradation is carried out by both extracellular phosphodiestarases, such as PDE, and intracellular ones, such as RegA or PdeE. The expression of these enzymes is regulated during development. In addition, their activity is regulated by extracellular signals, which allows precise control of intra- and extracellular cAMP levels through development [28].

Adenylyl cyclase A is a development-specific enzyme whose synthesis is induced during aggregation [29]. The generation of mutant strains has shown that *acaA* is required for this process [29]. This absolute requirement has impaired the study of the possible involvement of this enzyme during post-aggregative development, although *acaA* expression at the tip of early culminant structures has been described [17].

Adenylyl cyclase B, encoded by the *acrA* gene, is expressed at low levels in proliferating amoebas but its expression is strongly induced from 6 hours of development in prestalk cells [30], [31]. Mutant strains form normal structures up to the slug stage but *acrA* is required for culmination and terminal differentiation of the spores [31].

Expression of adenylyl cyclase G, encoded by the *acgA* gene, is induced after 12 hours of development in prespore cells and greatly increased during spore differentiation [29], [32]. AcG activity is regulated by osmolarity of the external media [22] and this enzyme is required to avoid spore germination inside the sorus. It has been recently described that AcG homologous enzymes play similar roles in other *Dictyostelium* species during cyst formation [33].

The analysis of the adenylyl cyclases described above indicates that the three enzymes play complementary roles during development. However, the present picture does not seem to be complete. Most of the developmental functions of the three enzymes have been deduced from the phenotype of single [29] [22] [31] or double mutant strains [32] and early developmental defects can preclude the observation of later ones, as can be the case for *acaA* gene mutants, that are blocked at aggregation. In addition, it has been shown that adenylyl cyclase genes regulate each others' expression [32], which could alter their function in mutant strains, as compared with wild-type ones. As a consequences, the function played by each or these enzymes in processes such as prespore and prestalk differentiation, sorting of prestalk and prespore cells in the mound and tip formation is not well determined at the present time.

In this article the structure of the *acaA* promoter region has been studied, showing that this gene is transcribed from three alternative promoters. The use of alternative promoters specific for different developmental stages or cell types has been previously described for some *D. discoideum* genes [34], [35], [36]. In the case of the *acaA* gene, the existence of alternative promoters allows expression during aggregation and. later on, in the tip-organiser and posterior regions of the structures. These results indicate that the spatio-temporal pattern of *acaA* expression is broader than previously considered and suggest the possibility that *acaA* might be involved in the regulation of several developmental processes, in addition to its well known role in aggregation.

## Methods

### Cell culture, transformation and development

*D. discoideum* cells were cultured in HL-5 media. Cells were transformed by electroporation as described by Pang et al. [37]. Transformed cells were selected by treatment with neomycin (G418). Filter development was induced by spreading 0.6-1.2×10^6^ cells/cm^2^ on Nitrocellulose filters (Millipore Co., Badford, MA, USA) [38].

### Rapid amplification of cDNA ends

RNA was isolated from AX4 cells at proliferation or after 8 hours of multicellular development. The SMART™ cDNA amplification kit from Clontech (Clontech Laboratories, Inc, Montain View, CA, USA) was used for amplification of the 5′ untranslated region of the *acaA* mRNA according to the manufacturer's instructions. The oligonucleotide 5′-GGAGATCTACCACCACCATTTCCATCATG-3′, complementary to nucleotides 90 to 110 of the *acaA* coding region, was used as primer in these experiments. Amplification products were cloned in the pGEMT-Easy cloning vector (Promega Co, Madison, WI, USA) and the insert of at least 10 different colonies of each product were sequenced.

### Construction of reporter vectors

The three *acaA* promoter regions were amplified by PCR from *D. discoideum* genomic DNA and cloned in the reporter vector PsA-ialphaGal [39] in substitution of the XbaI/BglII PsA promoter fragment. Oligonucleotides 5′-GGTCTAGACTTGATGAGTGGCCAAAACC-3′ and 5′-GGAGATCTATTTTTTAAAGATCCAAGAATTCGTATC-3′, that amplified the -3990 to -2472 genomic region, were used to isolate promoter 1 region. The antisense oligonucleotide included and ATG initiation codon cloned in frame with the *lacZ*-coding region. Oligonucleotides 5′-GGTCTAGAGTTTTTAGATACGAATTCTTGGATC-3′ and 5′-GGAGATCTCATTTACAAAGATATATTTATGAAGTGAGG-3′ amplified the −2500 to −1483 genomic region corresponding to Promoter 2. An ATG in frame initiation codon was also included in the antisense oligonucleotide. Oligonucleotides 5′-GGTCTAGACCTCACTTCATAAATATATCTTTG-3′ and 5′-GGAGATCTACCACCACCATTTCCATCATG-3′, that amplified the −1284 to 110 region were used to amplify promoter 3 region. This fragment included a region coding for the 37 N-terminal AcA aminoacids that were cloned in frame with the β-galactosidase protein. The complete promoter region was cloned in two steps. Promoters 1 and 2 were first cloned together using an internal XhoII site. To incorporate Promoter 3 to this construct a longer fragment (nucleotides −1838 to 110) was generated by PCR using oligonucleotides 5′-GGTCTAGAACCACATTTGTGTGAATTTGATTG-3′ and 5′-GGTCTAGACTTGATGAGTGGCCAAAACC-3′. This fragment was added to the Promoter1+promoter2 fragment using an internal NdeI site.

### Histochemistry and determination of β-galactosidase acivity

Cells transformed with the different reporter vectors were allowed to develop on Nitrocellulose filters for the periods or time indicated in each experiment. Structures were fixed, permeabilized and β-galactosidase activity was detected by hydrolysis of the X-Gal (5-Bromo-4-chloro-3-indolyl β-D-galactopyranoside) as previously described [40]. Spores were collected from structures developed on Nitrocellulose filters for 24 hours, fixed and permeabilized before detection of β-galactosidase activity as previously described [40]. β-galactosidase activity was also determined in extracts obtained at different developmental times. 2×10^7^ cells were developed on Nitrocellulose filters, collected and lysed in Z Buffer (60 mM Na_2_HPO_4_; 40 mM NaH_2_PO_4_, 10 mM KCl; 1 mM MgSO_4_, pH: 7.0) containing 0.2% NP40 (Nonidet P40). The enzymatic activity was determined by incubation of the extracts in Z Buffer containing 0.88 mg/ml of the ONPG (2-Nitrophenyl β-D-galactopyranoside) substrate. The amount of ONPG hydrolyzed was estimated by optical absorption at 410 nm and normalized to the amount of protein present in each sample.

### Determination of mRNA levels by quantitative RT-PCR

RNA was isolated from 2×10^7^ cells, either at growth or after development on Nitrocellulose filters for the times indicated in each experiment, using the TRI reagent (Sigma-Aldrich, Inc, St Louis, MO, USA) according to the manufacturer's instructions. cDNAs were generated from 1 µg of total RNA using gene-specific oligonucleotides as primers. cDNAs were used as substrates for quantitative real-time PCR reactions using as primers the oligonucleotides used for cDNA synthesis and a second oligonucleotide from the upstream region of each transcript. In the case of the acaA mRNAs, the oligonucleotide 5′-GGAGATCTACCACCACCATTTCCATCATG-3′, complementary to nucleotides 90 to 110 of the gene, encoded in Exon 2, was used for cDNA synthesis and as reverse primer for PCR amplification. The oligonucleotides 5′-CGTTTTTGATACGAATTCTTGGATC-3′ (nucleotides −2507 to −2483), 5′-CCTCACTTCATAAATATATCTTTG-3′ (nucleotides −1284 to −1261) and 5′-CTAGTAAAATTAATTTGTTGTACC-3′ (nucleotides −459 to −436) were used as forward primers for amplification of the cDNAs corresponding to mRNAs 1, 2 and 3, respectively. The oligonucleotide 5′-GGCATCTAGCTCACCAATG-3′ (nucleotides 3 to 21) was used as forward primer for amplification of a region of the cDNAs contained in Exon 2 that is common to the three mRNAs. A region of the large mitochondrial ribosomal RNA was amplified as a loading control using the oligonucleotides 5′-CACTTTAATGGGTGAACACC-3′ (used for reverse transcription and as reverse PCR primer) and 5′-GGGTAGTTTGACTGGGGCGG-3′ (forward PCR primer). The iQ5 Real Time PCR Detection System (Bio-Rad Lab. Inc., Hercules, CA, USA) was used in these experiments. PCR products were labeled with Sybr-green using the iQ™SYBR®Green Supermix (Bio-Rad) reaction mix following the manufacturer's instructions. The final volume of the reaction was of 20 µl, using a 0,16 µM concentration of each primer. PCR conditions were as follows: 95°C, 3 m; (95°C, 10 s; 58°C, 30 s; 68°C, 50 s)×40 for mRNA1 and Exon 2 expression; 95°C, 3 m; (95°C, 10 s; 60°C, 30 s; 72°C, 50 s)×40 for mRNA2 and 95°C, 3 m; (95°C, 10 s; 54°C, 30 s; 68°C, 50 s)×40 for mRNA3. The data, obtained in duplicates, were analyzed using the iQ5 Optical system software, version 2.0 (Bio-Rad).

### *In situ* hybridization and probe labelling

Whole-mount *in situ* hybridization of developmental structures was performed according to the method described by Escalante and Sastre [40] with minor modifications. Structures were developed on teflon® filters (Omnipore ™, Millipore Co., Badford, MA, USA), fixed and hybridized as described. 500 ng/mL of heat-denatured riboprobe were used for hybridization and colour reaction was stopped after 2 (streams) to 5 (culminants) hours. Pictures were taken (60X) with a camera (DFC420 Leica Microsystems, Wetzlar, Germany) attached to a stereo-microscope (MZ9_5_ Leica Microsystems).

Both sense and antisense RNA probes were prepared by *in vitro* transcription and digoxigenin labelling of the complete *acaA* ORF (kindly supplied by P. Schaap) using a DIG RNA labelling kit (Roche Diagnostics Mannheim, Germany) according to the manufacturer's protocol.

## Results

### 1. Structure of the *acaA* gene promoter region

**Figure 1:**
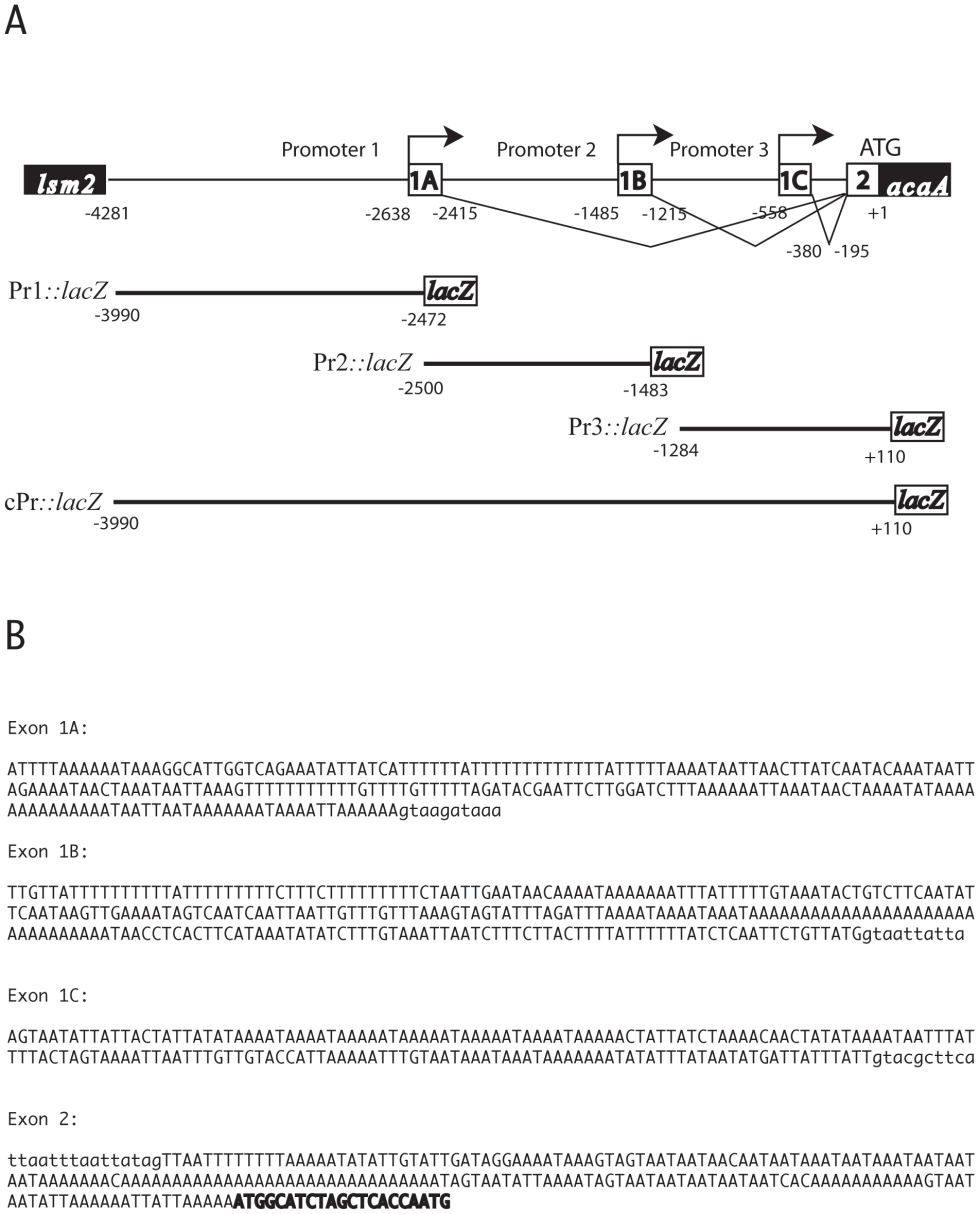
Structure of the *acaA* gene promoter region. A. The 5′ untranslated regions of three different *acaA* mRNAs were identified by rapid amplification of cDNA ends and their nucleotide sequences compared to that of this region of the genome. The results are schematically shown in the upper panel where transcribed exon regions are indicated as boxes. Protein coding regions are indicated as black boxes and untranslated regions as open boxes. The upstream gene closest to *acaA*, *lsm2*, is located to the left of the scheme. The three promoter regions identified, Promoters 1, 2 and 3, are labeled in the upper part and transcription initiation sites indicated by arrows. Splicing events that generate the three different mRNAs are indicated with thin lines in the lower part of the scheme. The position of transcription initiation sites and exon limits is shown underneath and numbered in relation to the initiation codon. The lower part of the figure schematically shows the promoter fragments that were cloned in the Psa-ialphaGal reporter vector for functional analysis: Pr1::*lacZ*, Pr2::*lacZ*, Pr3::*lacZ* and the complete promoter, cPr::*lacZ*. Numbers relate to the ATG initiation codon. B. Nucleotide sequence of the three alternative first exons identified, Exons 1A, 1B and 1C, and the common second exon, Exon 2, as determined from the products of Rapid Amplification of cDNA End reactions. Exon sequences are shown in capital letters and adjacent intron sequences, obtained for the genome sequence, in small letters, showing the presence of conserved donor and acceptor splicing sites. The Exon 2 protein-coding region is shown in bold face characters.

The study of the structure of the promoter region was initiated by determining the transcription initiation site by primer extension using the rapid amplification of the 5′ cDNA ends (RACE) method. Several amplification products were obtained using RNA from growing cells and from structures isolated after 8 hours of development, which pointed to the existence of more than one transcription initiation site. Amplification products were cloned and their nucleotide sequence determined. The comparison of the nucleotide sequence of the RACE products (shown in Fig. 1) with that of the *D. discoideum* genome indicated that the *acaA* gene is transcribed from three different promoter regions distributed along the 4 kb long *lsm2*-*acaA* intergenic region. The location of the three alternative 5′-untranslated regions is schematically shown in Figure 1A. Each of the three promoters drove transcription of a different non-coding first exon of the mRNA (Fig. 1B). Splicing of the three different mRNAs joined these specific exons to a common second exon where the translation initiation codon is located (Fig. 1B). Therefore, the three different mRNAs code for the same AcA protein.

### 2. Cell-type specific activity of the *acaA* promoters

The existence of different promoters regulating the expression of one gene is often associated with complex patterns of expression. In many cases each of the alternative promoters is active in a particular cell type or at different stages of development or cell differentiation [35], [36], [41]. To ascertain if that could be the case for the *acaA* gene, the pattern of activity of each promoter during multi-cellular development was determined by histochemistry. A *lacZ* gene coding for short-lived β-galactosidase was used as reporter in these experiments. Each one of the three promoters, and the intergenic region including all three promoters, was cloned in the reporter vector, as schematically shown in Figure 1A. These constructs were transfected in *D. discoideum* cells and β-galactosidase activity determined at different developmental stages. Pools of transformed cells were used in these experiments to avoid possible differences due to clonal variations in plasmid copy number or integration sites.

**Figure 2:**
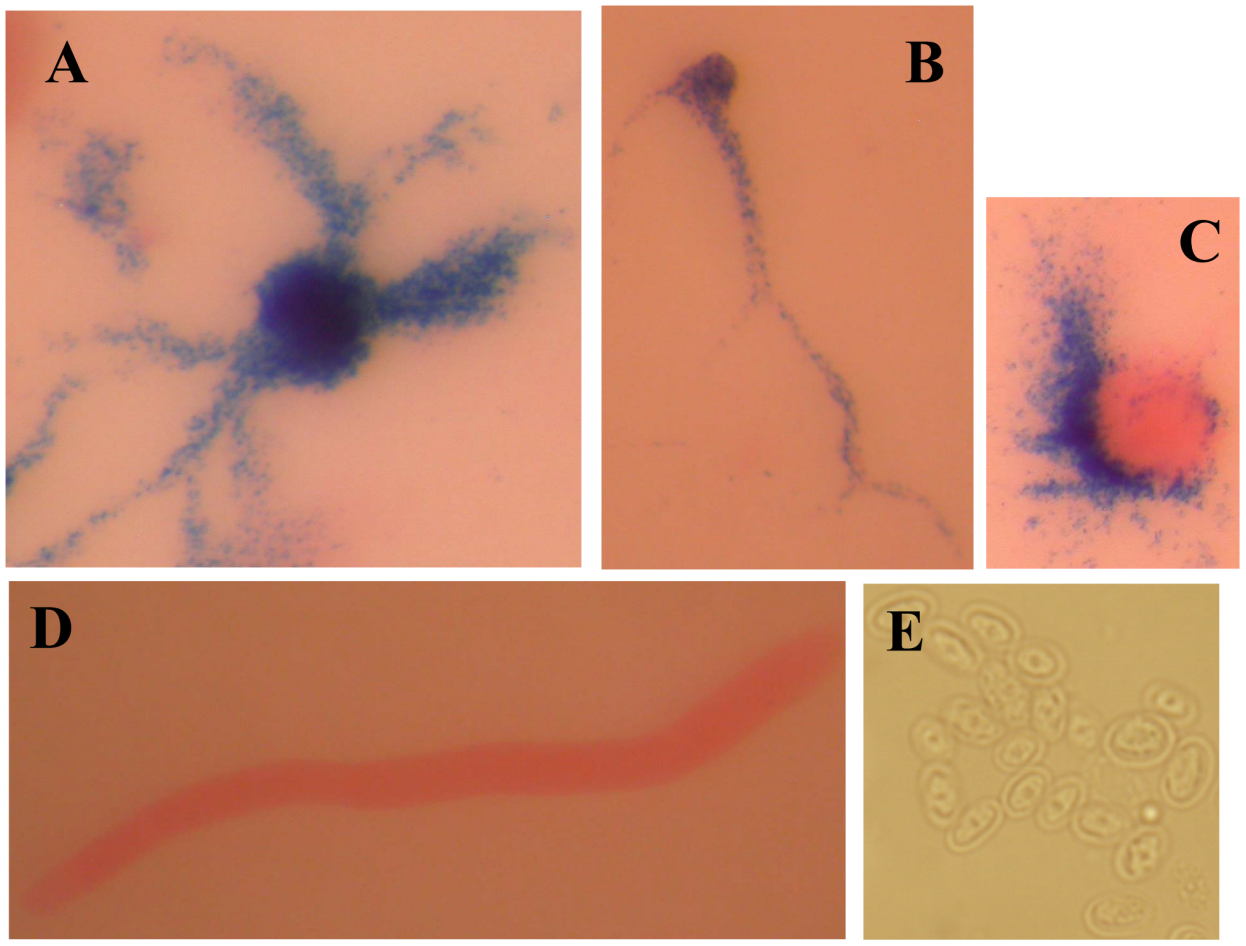
Activity of *acaA* Promoter 1 during *D. discoideum* development. *D. discoideum* AX4 cells were transformed with the reporter vector expressing the *lacZ* gene coding for short-lived β-galactosidase under the control of *acaA* Promoter 1. *lacZ* expression was detected by X-Gal hydrolysis and the structures were stained with eosine. Expression patterns obtained during cell aggregation (panels A and B), or at the early mound (C) and slug (D) stages of development and spores (E) are shown.

The histological pattern of β-galactosidase activity expressed under control of promoter 1 is shown in Figure 2. This promoter showed maximal activity during aggregation. Cells that are at the streaming stage of aggregation showed high levels of β-galactosidase expression (Fig. 2A,B). The activity of this promoter could be still observed in cells located at the base of tight aggregates (Fig. 2C) but was not detectable at later developmental stages, such as slug structures (Fig. 2D) or in spores (Fig. 2E).

**Figure 3:**
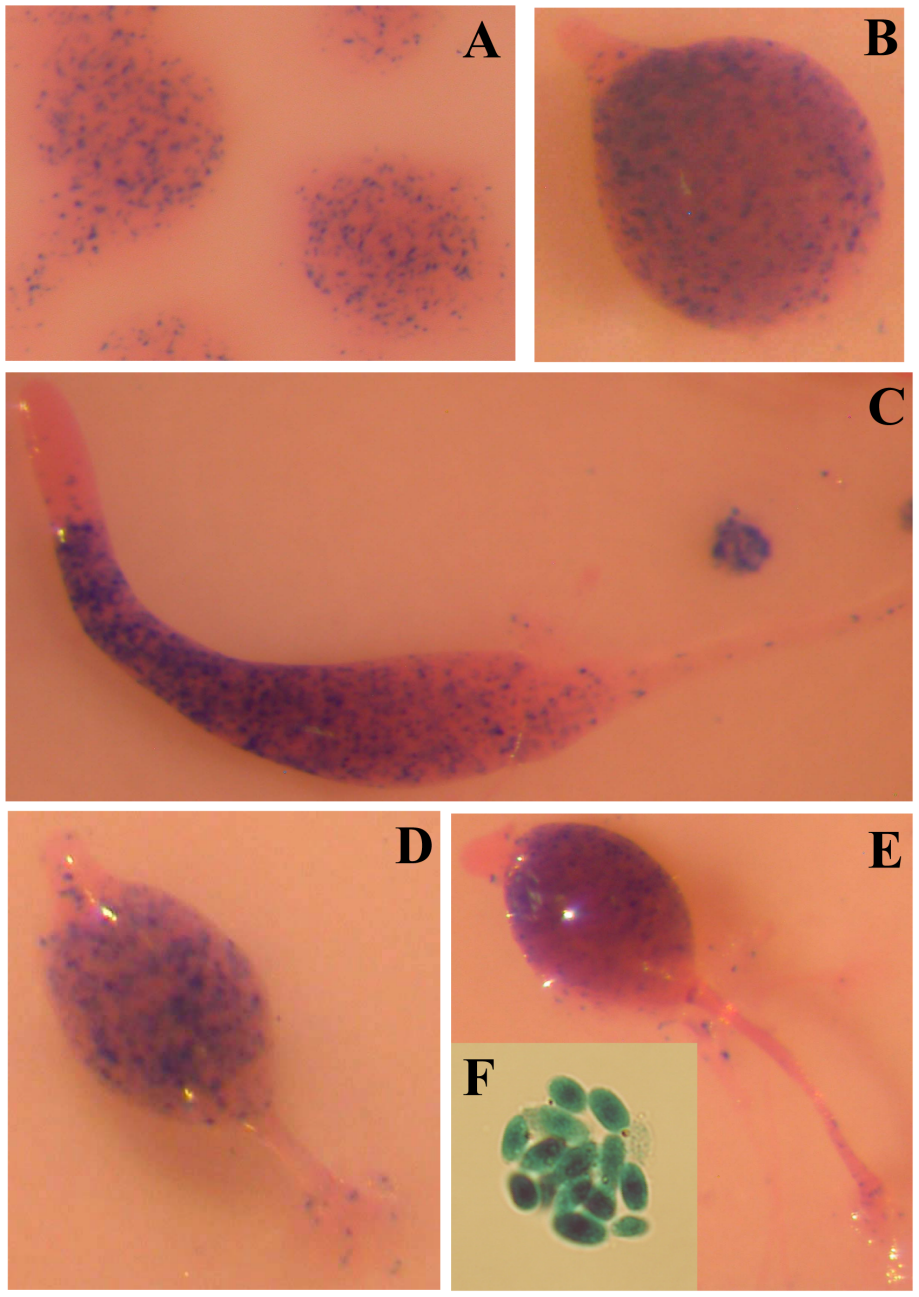
Activity of *acaA* Promoter 2 during *D. discoideum* development. AX4 *D. discoideum* cells were transformed with a reporter vector where the expression of a *lacZ* gene coding for short-lived β-galactosidase was under control of *acaA* Promoter 2. Transformed cells were allowed to enter multi-cellular development and *lacZ* expression determined by X-Gal hydrolysis at the early mound (panel A), tipped mound (B), slug (C), early culminant (D), late culminant (E) stages of development and spores (F). Structures were stained with eosine after X-Gal incubation.

Promoter 2 showed expression in cells dispersed through mound structures (Fig. 3A), and, later on, in cells located in the basal region of tipped mounds (Fig. 3B). The cells where Promoter 2 was active localized to the posterior region in slugs (Fig. 3C), with a pattern compatible with anterior-like cell- or prespore- specific expression, and to the mass of cells that migrate to the top of the structures during culmination (Fig. 3D, E). Expression was also observed in mature spores (Fig. 3F).

**Figure 4:**
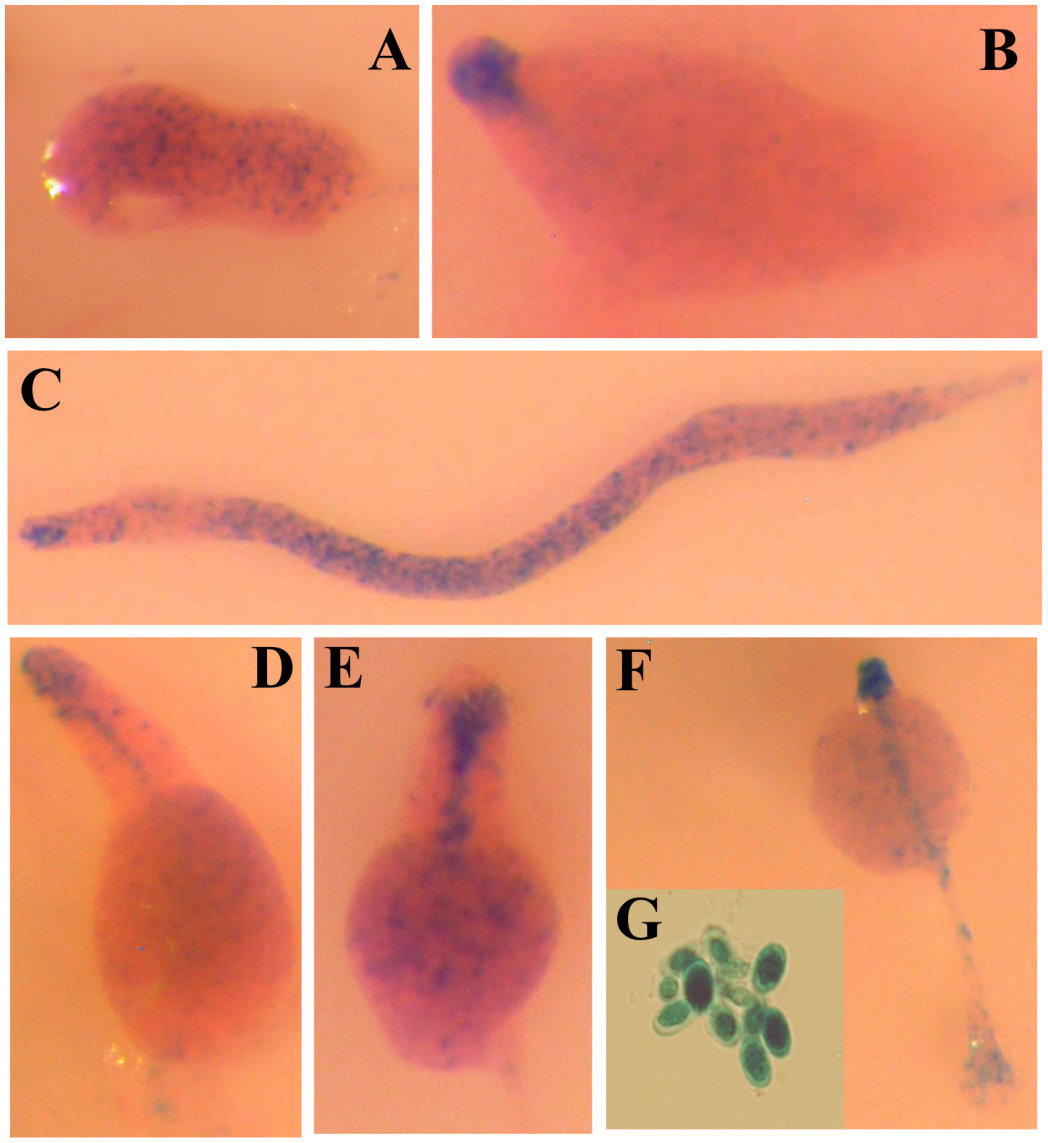
Activity of *acaA* Promoter 3 during *D. discoideum* development. *D. discoideum* AX4 cells were transformed with a vector where the reporter gene *lacZ*, coding for short-lived β-galactosidase, was under transcriptional control of *acaA* Promoter 3. *lacZ* expression was detected by X-Gal hydrolysis in the transformed strain at the early mound (A), finger (B), slug (C), early culminant (D), mid culminant (E), late culminant (F) stages of multi-cellular development and spores (G). Structures were stained with eosine after β-galactosidase detection.

The more proximal promoter, Promoter 3, was active in a group of cells with the characteristics of prestalk tip-organiser cells and in prespore cells (Fig. 4). Expression was initially observed in cells dispersed throughout mound structures (Fig. 4A). At the first finger stage, Promoter 3 was active in cells at the tip of the structure (Fig. 4B). In slugs Promoter 3-expressing cells were found in the anterior part of the structure with a pattern similar to that of tip-organiser cells, and in cells scattered through the posterior part of the structure (Fig. 4C). During culmination, Promoter 3-expresssing cells were located in the tip of the structure and in the stalk that was formed from the tip, towards the substrate (Fig. 4D, E). This pattern of staining was maintained until the last stages of culmination (Fig. 4F). In addition, a weaker staining was observed in prespore cells (Fig. 4E.F) and in mature spores (Fig. 4G).

**Figure 5:**
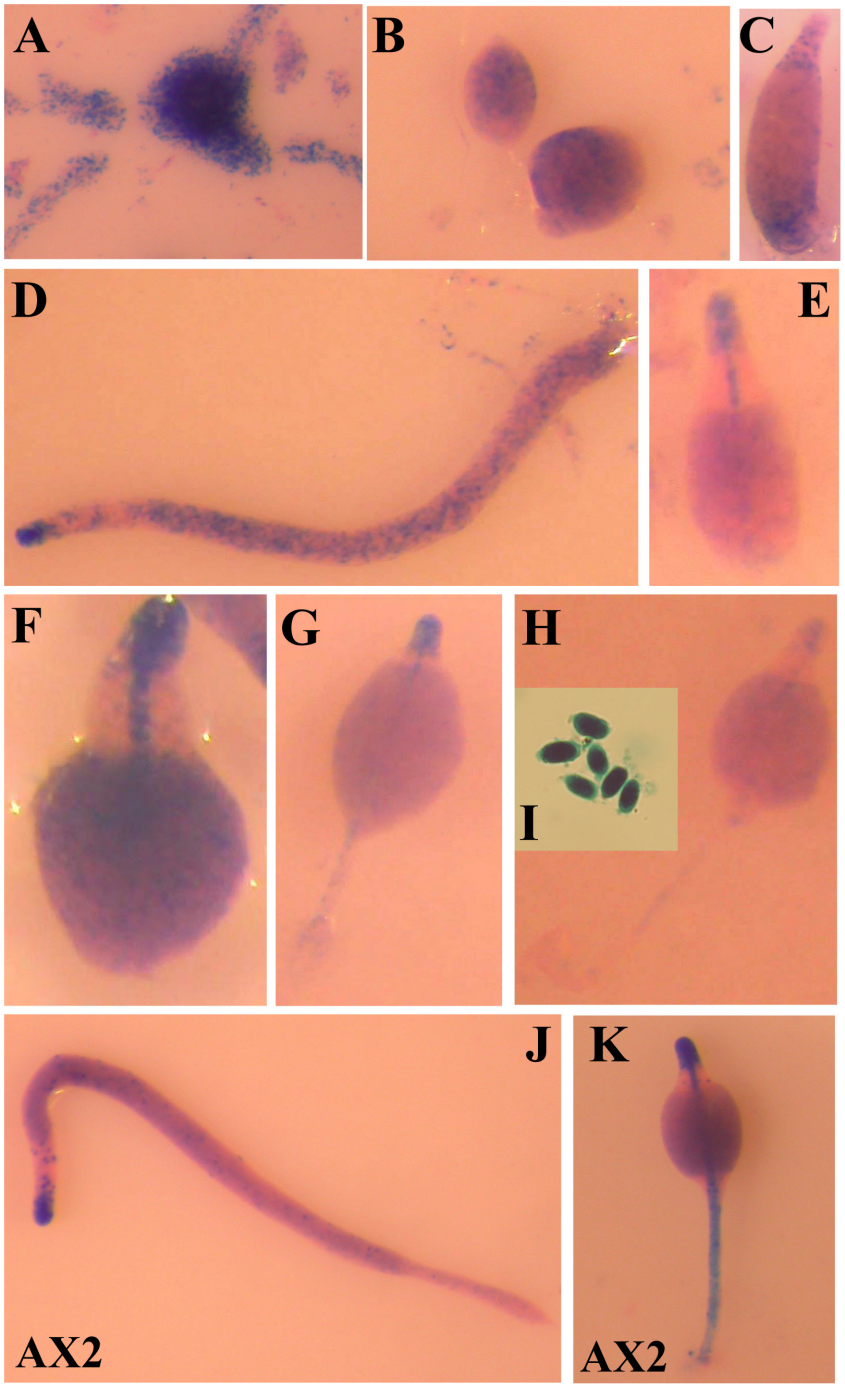
Activity of the complete *acaA* promoter during *D. discoideum* development. AX4 cells were transformed with a reporter vector where the complete *acaA* promoter region, covering most of the *lsm2*/*acaA* intergenic region, and including Promoters 1, 2 and 3, drives expression of a *lacZ* gene coding for short-lived β-galactosidase. Transformed cells were starved and *lacZ* expression determined during aggregation (panel A), at the mound (B), finger (C), slug (D), early culminant (E, F), mid culminant (G) and late culminant (H) stages of development and in spores (I). The same vector was also transformed in *D. discoideum* AX2 cells and *lacZ* expression determined at the slug (J) and late culminant (K) stages of multi-cellular development. Structures were stained with eosine after the determination of β-galactosidase activity.

The combined activity of the three promoter regions was studied by cloning most of the intergenic region from the closest upstream gene (*lsm2*) to the *acaA* second exon in the same reporter vector used above. The complete promoter (cPr) showed a pattern of cell-type specific activity corresponding to the addition of the three individual promoters (Fig. 5). The complete promoter drove β-galactosidase expression at aggregation (Fig. 5A) and in cells evenly distributed in the mound (Fig. 5B) and tipped-mound structures (Fig. 5C). At the slug stage and during culmination maximal staining was observed in tip-organiser cells, located to the tip of the structures, and in the stalk (Fig. 5D-H). However, β-galactosidase activity was also observed in the prespore region of slug (Fig. 5D), mid-culminant (Fig. 5E,F) and late culminant (Fig. 5G, H) structures, and in spores (Fig. 5I). To ascertain these results the reporter vector containing the complete promoter was transfected in a different *D. discoideum* axenic strain, AX2 cells. The pattern of β-galactosidase expression observed was similar to that of AX4 cells. Although tip-organiser cells showed maximal expression, staining at aggregation and in the prespore region of slug (Fig. 5J) and culminant structures (Fig. 5K) was also observed.

### 3. Temporal pattern of promoter activity

**Figure 6:**
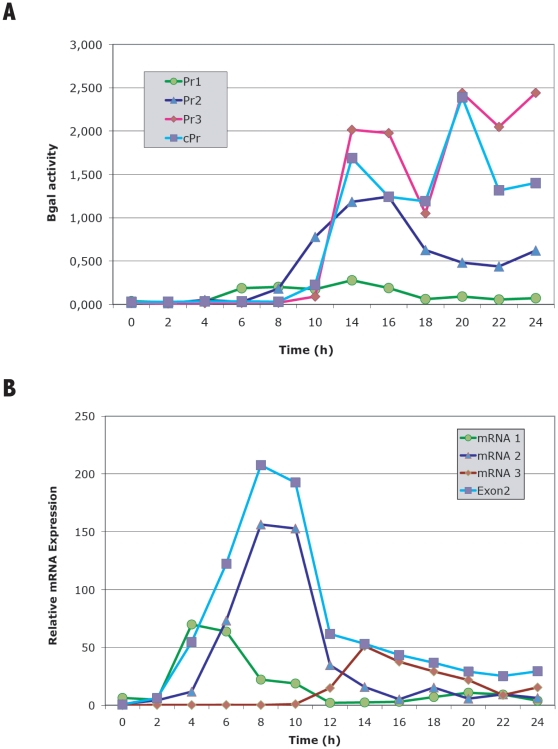
Analyses of acaA promoters activity and mRNAs expression during *D. discoideum* development. Panel A. *D. discoideum* AX4 cells were transformed with the reporter vectors where expression of a *lacZ* gene coding for short-lived β-galactosidase was under transcriptional control of *acaA* promotes 1, 2, 3 or the complete promoter. Transformed cells were allowed to develop on Nitrocellulose filters for the indicated hours (0–24). Collected samples were lysed and β-galactosidase activity determined using ONPG as substrate. A representative experiment where β-galactosidase activity was normalized by the amount of protein present in each sample is shown. (λ) Pr1::lacZ activity; (σ) Pr2::lacZ activity; (υ) Pr3::lacZ activity; (ν) cPr::lacZ activity. Panel B. RNA was extracted from growing cells (time 0) or from cells allowed to develop on Nitrocellulose filters for the indicated times (times 2–24). Expression of the different acaA mRNAs was analyzed by quantitative RT-PCR using oligonucleotides specific for the 5′ region of the three mRNAs detected in the RACE analysis (mRNA-1 λ, -2 σ and -3 υ). Oligonucleotides that amplify an exon 2 region common to the three mRNAs were used to estimate total acaA mRNA expression (Exon 2 ν). Relative expression levels, derived form the cycle when amplification is first detected, are indicated. These levels have been multiplied by a factor of 5 for mRNA1 and of 100 for mRNA3, in relation to mRNA2 and Exon2 levels.

The temporal pattern of activity was analyzed during development for each of the promoters by measuring β-galactosidase activity in cell extracts and the results are shown in figure 6A. In structures developed on Nitrocellulose filters, Promoter 1 was induced between 4 and 6 hours of development and remained active until 16 hours. Promoter 2 was induced between 6 and 10 hours of development and reached maximal activity between 14 and 16 hours to decrease thereafter. Promoter 3 was activated after 10 hours of development to remain active during the rest of the developmental process. The complete promoter was active from 6 hours of development although the main induction occurred between 10 and 14 hours, to reach a constant level of activity thereafter.

The activity of acaA promoters was compared to mRNA levels, estimated by quantitative RT-PCR. Oligonucleotides specific for each of the three alternative first exons were designed and used in conjunction with a reverse oligonucleotide complementary to a region of the second exon that is common to all the mRNAs detected previously. This approach allowed specific detection of the mRNAs transcribed from promoters 1 (mRNA1), 2 (mRNA2) and 3 (mRNA3). Total acaA mRNA was detected using oligonucleotides that amplified a region of the common exon 2. Induction of mRNA1 and mRNA3 during development correlated well with the pattern of Promoter 1 and 3 activation, respectively. However, mRNA2 was induced before any increase in Promoter 2 activity could be detected, indicating that regulatory regions not present in this promoter region could regulate mRNA2 expression. mRNA1 and mRNA3 steady-state levels were lower than those of mRNA2. This difference was especially significant for mRNA3, which could be due to the very localized expression of this mRNA at the tip-organiser region. The levels of total mRNA expression (Exon2) correlated well with the added expression of the three specific mRNAs.

### 4. Analysis of *acaA* mRNA expression by in situ hybridization

**Figure 7:**
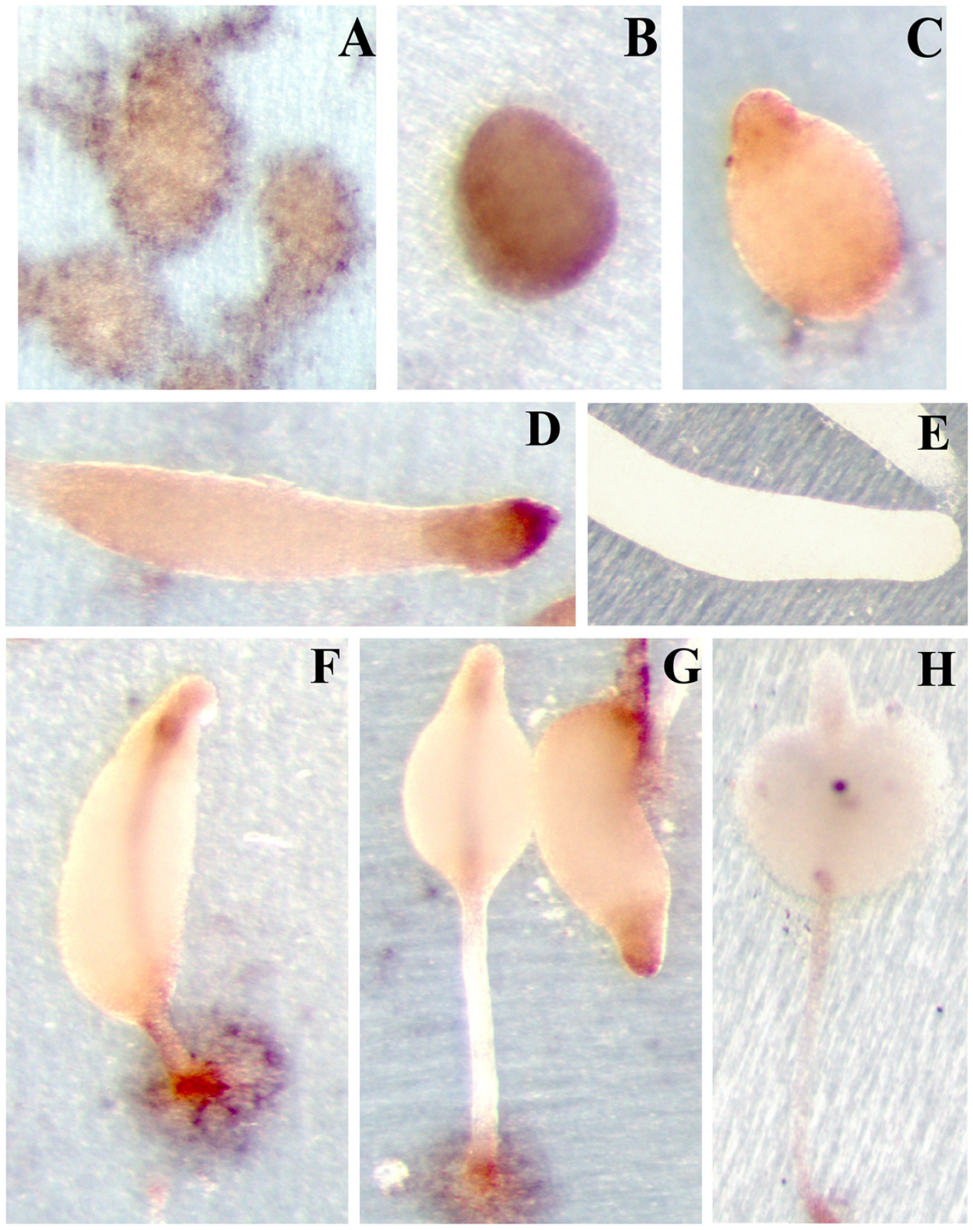
In situ hybridization analysis of *acaA* mRNA expression during *D. discoideum* development. *D. discoideum* AX4 cells were allowed to enter multi-cellular development on teflon® filters. Structures at the early aggregate (panel A), late aggregate (B), tipped mound (C), slug (D, E), early (F) and late culminant (G, H) stages of development were collected and *acaA* mRNA expression analyzed by in situ hybridization using an antisense RNA probe (A, B, C, D, F, G), or a sense RNA probe (E, H), as a control.

The results obtained in the study of the promoter region were compared to the analysis of mRNA expression by in situ hybridization. Antisense and sense RNA probes specific for the *acaA* mRNA were generated and used for in situ hybridization of structures at different developmental stages (Fig. 7). Intense, scattered hybridization was first observed during early aggregation and in mounds (Fig. 7A, B). Later on, strong specific hybridization was observed at the prestalk and/or stalk region of tipped mounds (Fig. 7C), slug (Fig. 7D) and early culminants (Fig. 7F,G). We could also observe moderate but specific hybridization in the prespore region of tipped mounds and slugs (Fig. 7C, D) and very week hybridization in the prespore region of early culminant structures (Fig. 7F, G). Incubation with a sense *acaA* RNA probe showed no specific hybridization (Fig. 7E,H). Both AX2 and AX4 developmental structures were analyzed by in situ hybridization and gave identical expression patterns.

## Discussion

Primer extension analysis indicates that the *acaA* gene is transcribed from three alternative promoters, although the three mRNAs that are generated code for the same protein. Functional analysis indicates that the more distal, Promoter 1, is specifically active in aggregating cells. Promoter 2 is active in aggregates and in the posterior region of developmental structures, while the most proximal promoter, Promoter 3, is mainly active in prestalk cells. Promoter 3 activity is not detected in all the cells of the prestalk region but only in the more anterior ones, including the previously described tip-organiser region. The study of the complete promoter showed an integrated pattern of expression that included all three promoters and displayed a general profile more similar to Promoter 3.

The results obtained with the promoter analysis were supported by the in situ hybridization experiments and by the temporal patterns of expression observed for the three *acaA* transcripts. Quantitative RT-PCR experiments using oligonucleotides specific for each of the 5′-untranslated regions were used to specifically measure the level of expression of each one or the three mRNAs. The results obtained indicated that the three mRNAs are expressed at different times during development. The mRNA transcribed from promoter 1 (mRNA1) was induced early during development to be repressed a few hours later, as also shown for promoter 1 activity. Induction and repression of the mRNA slightly preceded the observed variations in promoter activity. The temporal correlation between Promoter 3 activity and mRNA3 expression was fairly close with a marked increase between 10 and 14 hours of development to reach a plateau thereafter. More significant differences were found between the temporal pattern of Promoter 2 activity and mRNA2 expression, which is the more abundant and was induced six hours earlier than the observed increase in promoter activity. This result could be due to differences in the sensitivity of the detection method: Quantitative RT-PCR versus histochemistry. A second explanation could be the existence of interconnection between regulatory elements located in the different promoter regions analyzed. The experimental separation of the promoter in three regions according to the three transcription start points observed, which give the three alternative first exons, could be an oversimplification, even though they have proven to contain enough regulatory elements to regulate expression in different developmental structures. These promoter regions probably do not function independently and it could be expected that regulatory elements located in one promoter region affect the function of the others. In that case, β-galactosidase activity from the reporter constructs could differ from mRNA expression to some degree. That could be the case for Promoter 2 and mRNA2 where the early expression of the mRNA could be regulated by elements located outside of the Promoter 2 region analyzed. Interaction between regulatory elements located in different promoter regions could also explain the above-mentioned small differences observed between promoter 1 activity and mRNA1 expression.

Differences in the stability of *lacZ* and *acaA* mRNAs could also explain some of the discrepancies observed between the temporal patterns of promoter activity and mRNA levels during development. The three *acaA* mRNAs, and total mRNA, show sharper patterns of expression than those observed for promoter activity. mRNA steady-state levels increase and decrease before the corresponding levels of β-galactosidase activity. Given that a short-lived form of β-galactosidase has been used in these experiments, the differences could be explained if *acaA* mRNAs have a half-life shorter than *lacZ* mRNA. This difference seems to be especially significant at late developmental stages. Both Promoter 3 and the complete promoter are very active between 14 and 24 hours of development, as determined by β-galactosidase activity. However, *acaA* mRNA3 expression is maximal at 14 hours but decays markedly thereafter. The same decay is observed in total *acaA* mRNA steady-state levels, as also observed previously [29]. Since Promoter 3 activity is detected at the same developmental stage when mRNA3 starts to be expressed, these data could be explained by strong differences in *lacZ* and *acaA* mRNA stability.

Two studies on the *acaA* promoter region have been published previously. Verkerke-van Wijkt and collaborators [17] characterized an *acaA* promoter region isolated from a genomic clone. The comparison of the nucleotide sequence of this promoter and that of the genome indicates that the genomic clone contained an internal deletion that included part of Promoters 2 and 3 so that the promoter region studied was formed by a region of Promoter 2 (nucleotides −1838 to −1310) fused to the more proximal part of Promoter 3 (nucleotides −86 to 1). This promoter was active in cells at the mound stage and in tip-organiser cells, with a pattern of expression similar to the one shown for Promoter 3. These results could indicate the existence of a regulatory region activating gene expression at tip-organiser cells between nucleotides −86 and 1, that are also present in the Promoter 3 region analyzed in this article.

In addition Siol et al. [42] have characterized the promoter activity of a 773 bp fragment corresponding to the proximal region of Promoter 3 (nucleotides −739 to 34). This fragment activated transcription in aggregates and was dependent on the transcription factor CbfA. These authors also showed that CbfA was required for *acaA* expression during aggregation [43]. Since Promoter 1 is aggregation-specific, it will be of interest to determine if this promoter, that presents several possible binding sites for this transcription factor, is also dependent on CbfA for activation.

The expression of the *acaA* gene in prespore and prestalk cells in mound structures could be functionally relevant. An important difference between the three adenylyl cyclases is that their activity is regulated by different extracellular signals. Adenylyl cyclase A is homologous to G-protein coupled enzymes and its activity is regulated by G-protein coupled receptors through small G proteins [29], [44]. cAMP receptors, such as Car1 that mediate the response to extracellular cAMP during aggregation belong to this family of proteins. During aggregation the presence of extracellular cAMP induces cAMP synthesis by AcA through Car1, establishing a feed-forward loop that is essential for cAMP signaling [45]. *acaA* mutant cells do not aggregate but the addition of extracellular cAMP or of 2′ deoxy cAMP, that does not activate protein kinase A, or the presence of wild type cells, enables aggregation of the mutant cells. Indeed, mutant *acaA* cells can complete development with the addition of extracellular cAMP suggesting that the main role of the encoded enzyme during development is extracellular cAMP production [46].

This regulatory capacity is unique to AcA, because the other two adenylyl cyclases are not activated by G proteins. AcG is an osmosensor molecule activated by high osmotic pressure of the extracellular media [22]. The mechanisms that activate AcB are presently unknown but it does not depend on G proteins [31]. Some domains of the AcB N-terminal region present similarity to proteins involved in two-component signaling pathways, indicating that its activity could be regulated by phosphorylation in response to extracellular signals.

As mentioned in the Introduction, despite detailed studies of single and multiple adenylyl cyclases mutants, there are still cAMP-dependent developmental processes where the respective contribution of these enzymes is not clear. Some of them are processes that occur between the acaA-dependent aggregation step [29] and culmination, that is dependent on acrA [31]. For example, the initiation of cell differentiation or the first morphogenetic processes of cell sorting and tip formation. *acrA* and *acgA* mutants can complete these developmental processes. *acrA* and *acgA* double mutants are defective in spore formation but still express significant levels of prestalk and prespore genes [32]. The contribution of *acaA* to these processes has been difficult to determine because *acaA* mutants are not able to complete aggregation. However, the observation that *acaA* is expressed in the mound and in the tip-organiser region of finger and slug structures might indicate that AcA could be involved in cAMP signaling during these developmental stages. For example, it has been shown that cAMP waves continue to be formed from the upper part of the mound directing migration during cell sorting [47]. The expression of *acaA* at these structures and the regulatory capacity of the AcA enzyme would be in agreement with its implication in cAMP signaling during the formation of the tipped mound and slug structures. Similarly, AcA could be involved in the synthesis of the extracellular cAMP required for the first steps of prespore differentiation. The decrease of *acaA* expression in the prespore region at later developmental stages and the data on the *acrA* and *acgA* mutants [32] would indicate that AcB and AcG could be more important for terminal spore differentiation.

The existence of a promoter region specifically active at the tip-organiser region can be of interest to better understand the regulation of culmination. Several elegant studies have contributed to establish a cAMP-dependent gene transcription cascade that regulates the initiation of culmination (reviewed in [3]). The process is initiated by activation of the STATa transcription factor by extracellular cAMP [17]. STATa induces expression of the CudA transcription factor [16] that consequently activates expression of tip-organiser genes such as expl7 [18]. Tip-organiser-specific expression of acaA could be the first step, necessary for extracellular cAMP synthesis that initiates this culmination-inducing cascade.

The presence of a distal promoter (Promoter 1) specifically active during aggregation might have interesting evolutionary implications. Schaap et al [48] have shown that Dictyostelids can be classified in four groups and that *D. discoideum* belongs to the only group that uses cAMP as a signaling molecule at aggregation [49]. In contrast, all Dictyostelids use cAMP as a signaling molecule for the last steps of multi-cellular development and cell differentiation [50]. Therefore, the regulation of aggregation by extracellular cAMP seems to be a recent adaptation of a group of Dictyostelids, including *D. discoideum*. Alvarez-Curto et al [50] found that this adaptation involved significant changes in the expression pattern of the *car1* gene, coding for the cAMP receptor involved in chemotaxis. Car1 is expressed at aggregation only in species that use cAMP as signaling molecule and this change was associated with the acquisition of a new distal promoter region specifically active during aggregation [41]. Another gene required for cAMP signaling, *pdsA* coding for a extracellular phosphodiesterase, also has an aggregation-specific distal promoter [51]. These data impelled Alvarez-Curto et al [50] to propose that the adaptation of Dictyostelids to the use of cAMP at aggregation had involved the acquisition of new transcriptional regulatory capacities through the incorporation of new promoter regions. In agreement with this hypothesis, *acaA* would be the third example of a gene involved in cAMP signaling that has an aggregation-specific distal promoter. The determination of the nucleotide sequence of the genome of more Dictyostelids will allow us to determine if this distal promoter is only present in species that use cAMP for aggregation.

In summary, the observation that *acaA* is transcribed from three different promoters, during aggregation and multicelular development indicates that this gene can be involved in more developmental processes than the previously known aggregation step. The identification of these promoter regions makes possible to approach the study of the mechanisms that regulate *acaA* expression at the different developmental stages. In addition, the study of the contribution of *acaA* to the different developmental processes can be approached by complementation studies using each of the specific promoters.
